# Endocytosis of Corn Oil-Caseinate Emulsions In Vitro: Impacts of Droplet Sizes

**DOI:** 10.3390/nano7110349

**Published:** 2017-10-26

**Authors:** Yuting Fan, Yuzhu Zhang, Wally Yokoyama, Jiang Yi

**Affiliations:** 1 College of Chemistry and Environmental Engineering, Shenzhen University, Shenzhen 518060, China; fanyutingca@gmail.com; 2 Western Regional Research Center, Agricultural Research Service (ARS), United States Department of Agriculture (USDA), Albany, CA 94710, USA; Yuzhu.zhang@ars.usda.gov (Y.Z.); wally.yokoyama@ars.usda.gov (W.Y.)

**Keywords:** droplet size, Nile red, endocytosis, Caco-2, transport, nanoemulsion

## Abstract

The relative uptake and mechanisms of lipid-based emulsions of three different particle diameters by Caco-2 cells were studied. The corn oil-sodium caseinate emulsions showed little or no cytotoxicity even at 2 mg/mL protein concentration for any of the three droplet size emulsions. Confocal laser scanning microscopy (CLSM) of Nile red containing emulsions showed that the lipid-based emulsions were absorbed by Caco-2 cells. A negative correlation between the mean droplet size and cellular uptake was observed. There was a time-dependent and energy-dependent uptake as shown by incubation at different times and treatment with sodium azide a general inhibitor of active transport. The endocytosis of lipid-based emulsions was size-dependent. The internalization of nanoemulsion droplets into Caco-2 cells mainly occurred through clathrin- and caveolae/lipid raft-related pathways, while macropinocytosis route played the most important role for 556 nm emulsion endocytosis as shown by the use of specific pathway inhibitors. Permeability of the emulsion through the apical or basal routes also suggested that active transport may be the main route for lipid-based nanoemulsions. The results may assist in the design and application of lipid-based nanoemulsions in nutraceuticals and pharmaceuticals delivery.

## 1. Introduction

Some nutraceuticals such as curcumin and beta-carotene, due to the low water-solubility, poor chemical stability, high melting point, and low bioavailability, make the direct incorporation into food systems difficult [1,2]. The use of modern encapsulation and delivery systems such as emulsions, hydrogels, coacervates, liposomes, et al. can improve this. Among these, oil-in-water (o/w) emulsions are ideal delivery systems that have already been shown to be effective to improve water-solubility and bioavailability, especially for those extremely hydrophobic nutraceuticals or pharmaceuticals (e.g., carotenoids) [3]. The encapsulated nutraceuticals can also be designed for controlled release at certain cells or organs with emulsion-based delivery systems [4]. For example, compared with native curcumin, the bioavailability of colloidal curcumin was 14-, 5-, and 9-fold improved in women, men, and all subjects, respectively [5]. The preparation of o/w emulsions is relatively easy and cost-effective.

However, in principle, emulsions are usually thermodynamically unstable delivery systems, especially for those colloidal conventional emulsions, because their equilibrium state is a two-phase immiscible liquid, toward which the droplets evolve by various mechanisms (coalescence and Ostwald ripening being the primary ones) [6]. The instability will lead to creaming, coalescence, flocculation, and aggregation [7,8]. Lipid-based nanoemulsion may be an alternative for its higher stability, and higher bioavailability. Nanoemulsion is heterogeneous mixture of oil dispersed in water, where the oil droplet is confined to nanometer size (typically less than 200 nm) [9].

In the last decade, lipid-based nanoemulsions have been extensively studied and used as an excellent carrier for nutriceuticals [2,10,11]. Nanoemulsions not only have greater physical stability and protect nutrient from degradation by environmental factors but also improve the uptake and transport of nanodroplets in cell monolayers, compared to conventional emulsion [12,13,14]. Our previous work demonstrated that the extent of lipolysis and beta-carotene (BC) bioaccessibility was positively correlated with decreases of emulsion droplet diameter [14]. Zheng et al. showed that 5-demethyltangeretin-loaded emulsions with smaller droplet size led to higher cellular uptake and stronger inhibition on cancer cells [15]. Alpha-eleostearic acid nanoemulsions showed maximum efficacy in protecting cells from oxidative damage against both endogenous and exogenous reactive oxygen species (ROS) in lymphocytes and hepatocytes as compared to conventional emulsions formulation [16].

Nano-sized particle delivery systems can be absorbed directly by cells because of their small size [17]. The cellular uptake of nanoemulsions may be through different pathways, such as macropinocytosis, clathrin-mediated endocytosis, caveolae-mediated endocytosis, and clathrin- and caveolae-independent endocytosis [18]. It was reported that siRNA-loaded lipid nanoparticles enter cells by both constitutive and inducible pathways in a cell type-specific manner using clathrin-mediated endocytosis as well as macropinocytosis [19]. The mechanism of uptake of lipid-based nanoparticles have been reported to vary appreciably due to differences in emulsifiers, interface characteristics, and particle size [20]. Detailed information about cellular uptake and transport mechanism of lipid-based nanoemulsions are currently unavailable, and more studies are required.

In this study, sodium caseinate (SC) was used as emulsifier due to its biocompatible, biodegradable, and non-toxic characteristics, compared to small synthetic surfactants. Three different droplet size emulsions were prepared and the stability under storage was evaluated. Given the limitation of using animal or human subjects, the use of in vitro Caco-2 cells with many functions of the small intestinal villus epithelium for assessing the bioavailability of emulsions-based delivery system could be a cost-effective alternative. Cytotoxicity and cellular uptake of the three lipid-based emulsions (conventional emulsions and nanoemulsion) were also analyzed with Caco-2 cells. Cellular uptake and transport mechanisms of nanoemulsions were further investigated using inhibitors of the different uptake pathways.

## 2. Materials and Methods

### 2.1. Materials

Corn oil was purchased from a local market (Albany, CA, USA). Sodium caseinate (Alanate 180) was purchased from Fonterra Co-operative Group (Auckland, New Zealand). Nystatin, 5-(*N*-Ethyl-*N*-isopropyl) amiloride (EIPA), phenylarsine oxide (PAO), Nile red, and FITC-dextran (MW 40 kDa) were purchased from Sigma-Aldrich (St. Louis, MO, USA) and used without further purification. Sodium azide was obtained from Fisher Scientific (Fair Lawn, NJ, USA). Dulbecco’s modified Eagle’s medium (DMEM) (containing 4.5 g/L D-glucose and GlutaMAX™), penicillin and streptomycin (100×), fetal bovine serum (FBS), TrypLE^TM^ Select, Hanks’ balanced salt solution (HBSS), and phosphate buffer solution (PBS) (10×) were purchased from GIBCO (Grand Island, NY, USA). Alexa Fluor 488 to mouse IgG and 4′,6-diamidino-2-phenylindole (DAPI) were purchased from Life Technologies (Carlsbad, CA, USA). Caco-2, a human epithelial colon adenocarcinoma cell line, was purchased from the American Type Culture Collection (Manassas, VA, USA). All other analytical grade chemicals and reagents were purchased from Fisher Scientific (Fair Lawn, NJ, USA). Ultrapure water was used in all experiments.

### 2.2. Preparation of Varying Droplet Size Emulsions

Sodium caseinate (SC) was dispersed in ultrapure water and stirred for 2 h to form a 2% solution. Nile red (1 mg/mL), a fluorescent dye, was dissolved in corn oil by stirring for 10 min at 25 °C in the dark. Crude o/w emulsions with a 10% volume fraction of the Nile red corn oil solution in the 2% SC solution was formed by high-speed homogenization for 2 min at 13,000 rpm (T25, IKA-Werk, Staufen, Germany). The crude emulsion was then further homogenized through a high pressure microfluidizer (M-110L, Microfluidics, MA, USA) seven times at two pressures, 9000 psi (62.1 MPa), and 15,000 psi (103.4 MPa), to yield two emulsions (No. 2 (265 nm), and No. 3 (170 nm)) of various droplet diameters at room temperature. Emulsion No. 1 (556 nm) was prepared by a 10% volume fraction of the Nile red corn oil in SC solution through the homogenizer (Niro-Soavi Panda, Parma, Italy) five times at 40 MPa. After the preparation, all stock samples were refrigerated (2–6 °C) for subsequent uses.

### 2.3. Droplet Diameter Analysis

The mean droplet sizes (*D*z), polydispersity indices (PDI), and Z-potential were determined by dynamic light scattering (Zetasizer Nano, Malvern Instruments, Worcestershire, UK). The three different mean droplet diameter emulsions were diluted 100 folds with distilled water, and adjusted to pH 7.0 with NaOH or HCl. The refractive index values used for the instrumental analysis of oil droplets and dispersant were 1.45 and 1.33, respectively. All measurements were made three times and were performeded at 25 °C.

### 2.4. Droplet Size Stability

The storage stability of the emulsions during storage (1 month) without light was analyzed by mean droplet sizes with Zetasizer Nano after storage for 30 days at room temperature.

For the cellular uptake stability, emulsions were diluted 10-fold in PBS (pH 7.4) and the mean droplet diameters were determined with Zetasizer Nano at certain time intervals (0, 0.5, 1, 2, and 4 h).

### 2.5. Transmission Electron Microscopy (TEM) Analysis

TEM was used to visualize the shapes and characteristics of lipid emulsions and to confirm the *D*z by dynamic light scattering. Samples were prepared by the conventional negative-staining method. Lipid-based emulsions were placed on carbon-coated copper grids and negatively stained with 2% (*w*/*v*) phosphotungstic acid for several minutes at room temperature, and dried in air. Grids bearing lipid emulsions were measured with a transmission electron microscope (Hitachi H-700, Tokyo, Japan).

### 2.6. Cytotoxicity of Lipid-Based Emulsions

The application of nanocarriers in food system may causes some concerns about the potential toxicity to human health. The potential cytotoxicity of the three different droplet size lipid-based emulsion system was tested referring to previously published method [21]. In brief, Caco-2 cells (between 120–130 passages) were seeded at a density of 1.0 × 10^5^ cells/well on 96-well plates and incubated at 37 °C and 5% CO_2_ in a humid atmosphere for two days (Sanyo, Osaka, Japan). Notably, the biological characteristics and phenotypes of Caco-2 cells will differ from high and low passage [22]. After that, Caco-2 cells were treated with nanoemulsion or conventional emulsions at different sodium caseinate concentrations (20, 2, 1, 0.5, and 0.2 mg/mL) for 4 h and then incubated with MTT (3-(4,5-dimethylthiazol-2-yl)-2,5-diphenyltetrazolium bromide) diluted in PBS (pH 7.4) for 2 h at 37 °C. DMEM was used as control. In addition, the absorbance was measured at 570 nm with 96-well plate microplate reader (Epoch, BioTek, VT, USA). Cell viability was evaluated by the percentage of absorbance relative to control.

### 2.7. Cellular Uptake

Caco-2 cells (ATCC) were grown on 25 mm glass coverslips and maintained in DMEM supplemented with 10% fetal bovine serum (FBS), 1% non-essential amino acids, 0.01 mg·mL^−1^ of human transferrin, 10,000 U of penicillin per mL, and 10 ng of streptomycin per mL in an incubator (Sanyo, Osaka, Japan) with an atmosphere of 95% air-5% CO_2_ according to previously described 24method [22]. The medium was changed every other day. After five days, the cell monolayers were observed with an optical microscope (Leica, IL, USA) to ensure that the confluence reached approximately 95%. Caco-2 cell monolayers were washed with PBS (pH 7.4) three times. Three different mean droplet diameter emulsions (2 mg sodium caseinate/m Lin PBS) were added to each well. Following 4 h incubation, the supernatants were removed and cell monolayers were washed three times with pre-cooled PBS solution to stop cellular uptake. Cells in coverslips were then fixed in 3.7% paraformaldehyde (PFA) for 20 min and rinsed with PBS (pH 7.4) three times before fluorescence staining.

### 2.8. Effects of Inhibitors on Cellular Uptake of Lipid-Based Nanoemulsions

Four different blocking reagents (Nystatin, EIPA, PAO, and sodium azide), with various inhibition mechanisms were used to analyze the specific mechanism of lipid-based nanoemulsions, which was involved in the cell uptake.

Caco-2 cells were pre-incubated with four blocking reagents at certain concentrations (Table 1) for 30 min, respectively, and then cells were incubated with nanoemulsions for 4 h at 37 °C for cell uptake experiment. The control was cells incubated PBS (pH 7.4) without inhibitors. The results were expressed as the inhibition percentage versus control.

In order to study the effects of temperature on nanoemulsions cellular uptake, Caco-2 cells were incubated with nanoemulsions at 4 and 37 °C, respectively. At certain intervals (0.5, 1, 2, and 4 h), cells were treated following the steps described in Cellular uptake, and photographed with CLSM and quantified with microplate fluorescence reader.

### 2.9. Confocal Laser Scanning Microscopy (CLSM)

Lipid-based emulsions cellular uptake was conducted based on confluent Caco-2 cells on glass coverslips. After cellular uptake, Caco-2 cells were rinsed three times with PBS, permeabilized with 1% triton X-100 in PBS for 30 min, and then incubated with blocking solution (0.2% Triton X-100 and 5% bovine serum albumin) for 1 h according to previous method with slight modification [23]. After washing thrice, cells were incubated with Alexa Fluor 488 bound to mouse anti-actin IgG (1:500 dilution; Life Technologies) and mounted onto glass slides using the nuclear counter stain DAPI incorporated in a hard-set mounting medium (Vector Laboratories). Fluorescent Nile red incorporated in the corn oil was excited with an argon laser at 488 nm. Caco-2 cells were observed with a Leica Microsystems confocal microscope (Leica TCS SP5, Wetzlar, Germany) with the appropriate filter sets. All experiments were performed in triplicate.

### 2.10. Quantification Study

Caco-2 cells (between 120–130 passages) were seeded at a density of 1.0 × 10^5^ cells/well of 96-well black plates and incubated until the confluence reached at least 95%. After uptake, cells were then treated with 0.1% Triton X-100 lysis buffer. In addition, the fluorescence intensity of Nile red was measured at excitation wavelength of 530/25 nm and emission wavelength of 590/35 nm with a microplate fluorescence reader (SpectraMax M3, Molecular Devices, CA, USA). A linear standard curve of fluorescence vs Nile red standard concentrations from 0.01–1 μg/mL (0.01, 0.02, 0.05, 0.1, 0.2, 1.0 μg/mL) was applied to determine the transport of lipid-based nanoemulsions. Nile red methanol dispersed in PBS was used as standard.

### 2.11. Transport Study

The transport of lipid nanoemulsions was studied by the incubation of Caco-2 monolayer in transwells following a recently reported protocol with slight modification [21,24]. In brief, Caco-2 cells were seeded at a density of 1.0 × 10^5^ cells/well onto a tissue culture-treated polycarbonate filter (Millipore, Billerica, MA, USA), with membrane area of 4.2 cm^2^, in 6-well plates. The medium was replaced every 48 h for the first week and every 24 h for the next two to three weeks. Transport experiments were performed between 21 and 28 days. Both the trans-epithelial electrical resistance (TER) value and the apparent permeation rate of the paracellular permeation marker (*P*_app_, FITC-dextran) were monitored to ensure the integrity of Caco-2 cell monolayers after incubation. For the transport experiments, the Caco-2 monolayers were washed with PBS (pH 7.4) three times. For apical to basolateral compartment transport, 1.5 mL of the lipid-based nanoemulsions diluted in PBS to a final Nile red concentration of 10 μg/mL were added to apical side. For basolateral to apical compartment transport, 2 mL nanoemulsions diluted in PBS (pH 7.4) (Nile red is 10 μg/mL) was added the basolateral side and 1.5 mL receiving medium was added to apical side. The medium in the basal side was sampled at designated time point during transport experiment, and detected with microplate fluorescence readers (SpectraMax M3). The fluorescence intensity of Nile red was measured at excitation wavelength of 530/25 nm and emission wavelength of 590/35 nm.

TER values was also measured at time intervals (0, 0.5, 1, 2, 3, and 4 h) to measure the integrity of Caco-2 monolayers.

The apparent permeability coefficient (*P*_app_, unit: cm/s) was calculated using the following formula:

(1) $$P_{\mathrm{app}}=(dQ/dt)(1/(AC_{0}))$$

where d*Q*/d*t* is the permeability rate (µg/s), *A* is the surface area of the filter (cm^2^), and C_0_ is the initial concentration in the donor chamber (µg/mL).

Before transport, after 3 weeks incubation, TER values were measured with an Evohm2 epithelial voltmeter (World Precision Instruments, Sarasota, FL, USA) and was above 350 Ω·cm^2^; and the *P*_app_ of 1 mg/mL FITC-dextran detected with a microplate fluorescence readers (SpectraMax M3) at excitation wavelength of 487 nm and emission of 518 nm was (5.3 ± 1.2) × 10^−8^ cm/s. Both the TER, which was higher than reported values (260 ± 65 Ω·cm^2^) [24] and *P*_app_, which was lower than reported values, indicated that the Caco-2 cell monolayers were confluent and suitable for permeation study.

### 2.12. Statistical Analysis

The data were expressed as mean ± standard deviation with three independent replicates. The data were subjected to the analysis of variance (ANOVA) with the SPSS 17.0 package (IBM, New York, NY, USA). Differences with a value of *p* < 0.05 were considered statistically significant.

## 3. Results and Discussion

### 3.1. Characteristics of Emulsions

**Figure 1:**
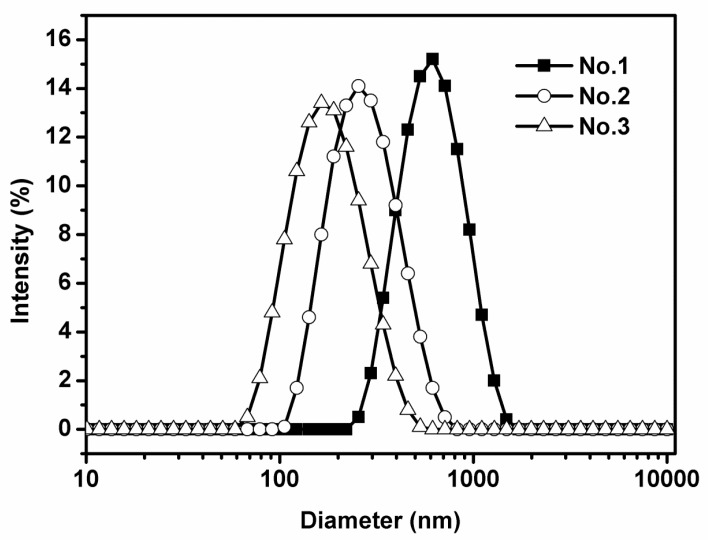
Droplet diameter distributions of three lipid-based emulsions with varying droplet size encapsulated with sodium caseinate (mean droplet sizes of No. 1, No. 2, and No. 3 are 556, 265, and 170 nm, respectively). The data were expressed as mean ± standard with three independent replicates.

**Figure 2:**
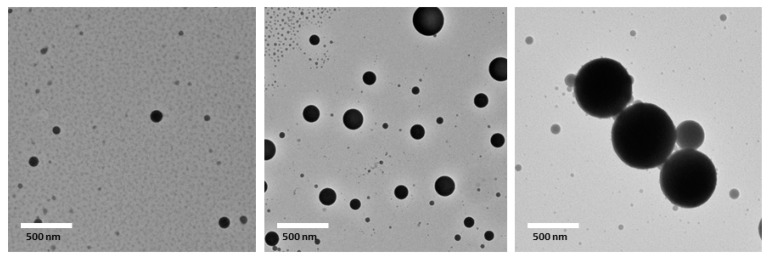
Transmission electron microscopy (TEM) images of three lipid-based emulsions (mean droplet sizes of No. 1, No. 2, and No. 3 are 556, 265, and 170 nm, respectively). All of the data were presented as mean ± standard deviation from at least three measurements.

Three emulsions with mean droplet diameters of 556, 265, and 170 nm, respectively, with the same oil phase and emulsifier were prepared by shearing with a microfluidizer or homogenizer to investigate the mechanisms of cellular uptake of nanoemulsions. As shown in Table 2, all PDI values were below 0.2, indicating uniform droplet size distributions. In addition, droplet size distribution were also unimodal (Figure 1). The zeta-potential of three emulsions were −41 to −43 mV. The similar zeta-potentials (*p* > 0.05) indicate that droplet sizes did not have a major impact on droplet surface charge (Table 2). The morphology of emulsions was observed by transmission electron microscope (TEM). All three emulsions contained spherically shaped and fairly uniform droplets with mean droplet diameters similar to the results obtained with dynamic light scattering (Figure 2). Noteworthily, a few of droplet sizes in the range of 10–50 nm is possible an artifact of the drying required for sample preparation. Since the color of the droplets (between 10 and 50 nm) is grey, which is significantly different with lipid droplets.

### 3.2. Cytotoxicity of Lipid-Based Emulsions

**Figure 3:**
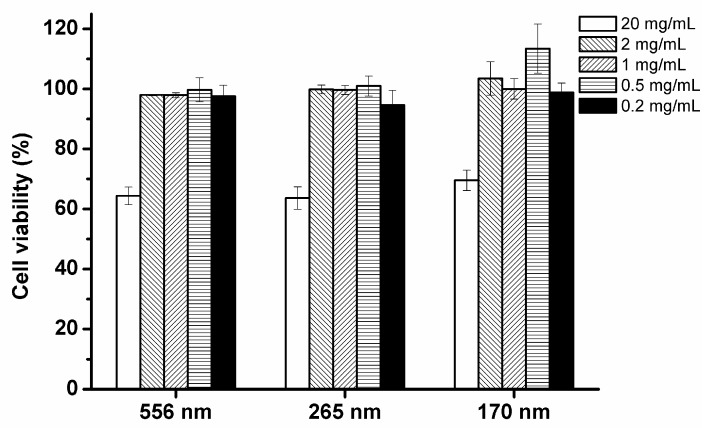
In vitro cytotoxicity of three different droplet size lipid-based emulsions at different sodium caseinate concentrations (20, 2, 1, 0.5, and 0.2 mg/mL) on Caco-2 cells measured by MTT assay. Cell viability was evaluated by the percentage of absorbance relative to control. The data were expressed as mean ± standard with three independent replicates.

The inhalation of nanoparticles has raised the possibility of toxicity even for nutraceutical nanocarriers formulated with food grade ingredients [17,22]. The potential cytotoxicity of the emulsions and droplet size effects were evaluated in a Caco-2 cell model with cell viability determined by MTT assay at varying dilutions (Figure 3). The results indicated that the viability of cells was dependent on the concentration of emulsions. For un-diluted emulsions (initial sodium caseinate concentration, C_0_ = 2% = 20 mg/mL, corn oil = 10% = 100 mg/mL) the cell viabilities relative to control were 64%, 64%, and 69% for 556, 265, and 170 nm, respectively. The results indicated that the 10% lipid-based emulsions are toxic without dilution and there were no noticeable size effects on cytotoxicity. After 1:10 dilution (2 mg/mL protein concentration and 10 mg/mL oil concentration), cell viabilities relative to DMEM control, were 98%, 100%, and 103% for 556, 265, and 170 nm, respectively, suggesting that the lipid-based emulsions showed no toxicity. All the cell survival rates were above 95% when treated with emulsions of 2 mg sodium caseinate/mL or less. The results indicate that the cytotoxicity of lipid-based emulsions are concentration-dependent. Lipid-based emulsions of 2 mg sodium caseinate/mL were used for further study of the cellular uptake mechanism.

### 3.3. Mean Droplet Diameter Stability of Lipid-Based Emulsions

After 30 d storage at room temperature, the mean droplet size of all three emulsions increased. All PDI values increased indicating the droplet size distributions have broadened (Table 2). Larger droplets rendered the largest increases in size. This is probably due to droplets flocculation or coalescence when sodium caseinate encapsulated droplets interacted with each other because of convection currents produced by temperature gradients or normal vibrations. The similar zeta-potentials (*p* > 0.05) indicate that droplet sizes did not have a major impact on droplet surface charge (Table 2). No visible sediments or aggregates were observed in the stored emulsion samples also suggesting that the emulsions were fairly stable during 30 d storage. These observations were similar to those of Dickinson et al. who showed that protein stabilized oil-in-water (o/w) emulsions produced by high pressure homogenization are stable when the droplet diameter is less than 1 μm [25].

**Figure 4:**
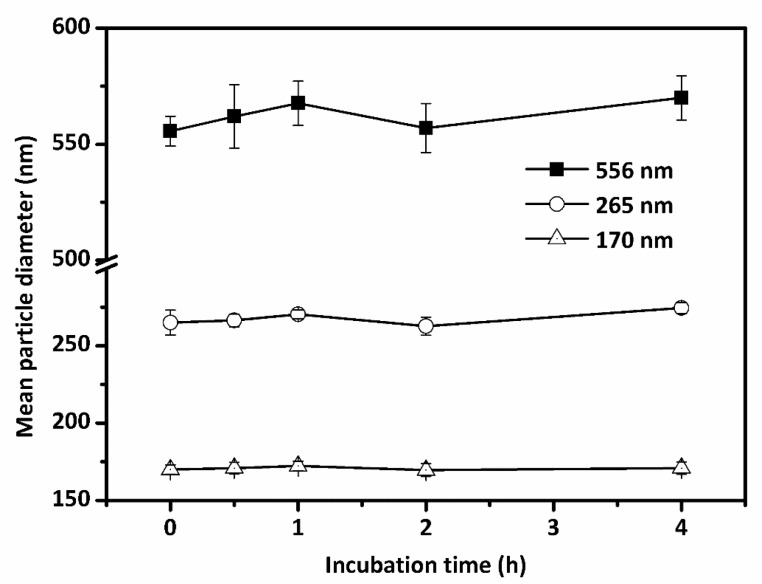
Stability of three different mean droplet size emulsions (556, 265, and 170 nm, respectively) during 4 h incubation in PBS (pH 7.4) at 37 °C. Stability was evaluated by the mean droplet diameter changes of emulsions measured at several incubation intervals (0, 0.5, 1, 2, and 4 h).

The three various droplet size emulsions of 2 mg sodium caseinate/mL in PBS (10 mM, pH 7.4) were used for stability tests during cellular uptake experiment. The emulsions were then incubated at 37 °C for 4 h, and the mean droplet size was measured at several incubation intervals (0, 0.5, 1, 2, and 4 h). As can be seen in Figure 4, the mean droplet sizes were increased to 570 and 274 nm for two conventional emulsions (556 and 265 nm), respectively after 4 h (*p* > 0.05). Almost no droplet size increase was observed for nanoemulsion (170 nm). Better stabilities of lipid-based emulsions were observed with smaller mean droplet size. The results clearly showed that SC coated lipid-based droplets can provide enough electrostatic repulsion of lipid-based droplets against aggregation and flocculation in PBS.

### 3.4. Effects of Droplet Size on Cellular Uptake of Lipid-Based Emulsions

Previous studies [12], including ours [22], have clearly indicated that emulsion is an ideal delivery system for improving the bioavailability of encapsulated nutraceuticals. In order to characterize endocytosis of emulsions, Nile red, a fluorescent lipid-dissoluble dye, was used as a probe.

**Figure 5:**
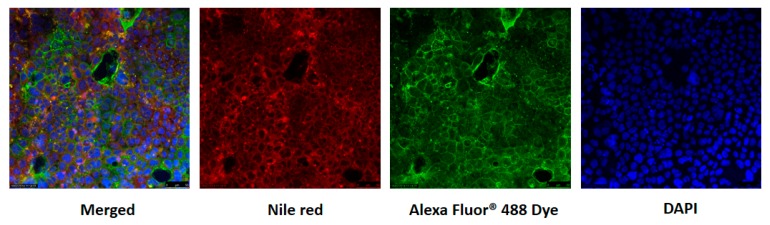
CLSM of Caco-2 cells grown on glass coverslips after exposure to lipid-based nanoemulsions (mean droplet size is 170 nm) for 4 h. Cell nuclei were stained in blue with DAPI; β-actin labelled with Alexa Fluor 488 appear as green spots; nanoemulsion lipid labeled with Nile red; first picture displays the merged (*x*,*y*) image of stains of Caco-2 cells after incubation with nanoemulsion. Scale bar indicates 15 μm. The data were expressed as mean ± standard with three independent replicates.

Endocytosis has been defined as the process by which nanoparticles or macromolecules are transported into cells by an energy consuming process [18]. There are three main endocytic pathways: clathrin-mediated endocytosis, lipid raft/caveolae-mediated endocytosis, and macropinocytosis. As illustrated in Figure 5, Caco-2 cells were incubated until confluent as shown by DAPI nuclear counterstain and Alexa Fluor 488 labeled β-actin that is suitable for uptake experiment. The Nile red incorporated in the corn oil showed that the lipid-based nanoemulsions were clearly internalized into Caco-2 cells, consistent with the recent published results [26]. The nanoemulsions in the cells labeled by Nile red are shown to be surrounded by β-actin labeled with green fluorescing Alexa Fluor 488.

**Figure 6:**
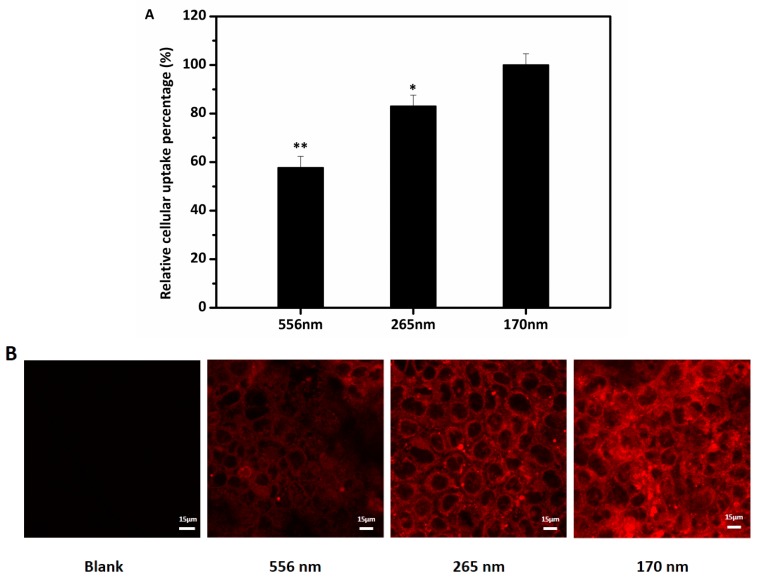
The effects of three different droplet sizes on cellular uptake of lipid-based emulsions (**A**) Relative uptake of emulsions of different diameters by Caco-2 cells. The 170 nanoemulsions were used as control (100% uptake). Other lipid emulsion sizes were compared to the control using ANOVA (* *p* < 0.05, ** *p* < 0.01). (**B**) CLSM of Caco-2 cells grown on glass coverslips after exposure to three different droplet size lipid-based emulsions containing Nile red for 4 h. Scale bar indicates 15 μm. The data were expressed as mean ± standard with three independent replicates.

Numerous studies [15,27,28] have indicated that increased cellular uptake and transport of encapsulated bioactive compound are linked to decreased droplet size, however, other factors including emulsifiers, carrier oil, interfacial characters, or loading amount represent significant confounding effects [29,30,31]. In our study, three emulsions with different droplet sizes (556, 265, and 170 nm) were prepared and used to evaluate the effects of droplet size on the uptake of lipid-based emulsions. As can be seen in Figure 6, the uptake represented by the fluorescence intensity was remarkably different for the three different emulsions. There was a positive correlation between reduced droplet size and increased cellular uptake. We observed in our previous study that smaller droplet size of lipid-based emulsions improved bioaccessibility [14]. Two possible mechanisms (size-dependent contact curvature and size-dependent droplet deformability) were proposed for this [32,33,34]. The decreases of droplet size increase the adhesive contacts between cellular membrane and droplet, thereby significantly decreasing the energy requirements for deforming the membrane around the droplets, which led to the increase of cellular uptake [33,34]. Furthermore, the decreases of deformability of the droplets due to the decrease of droplets size may also resulted in the increase in cellular internalization [32,34]. Other studies have also reported similar results, for example, the absorption rate of gold nanoparticles was negatively correlated with the mean particle size [28].

### 3.5. Effects of Four Inhibitors on the Cellular Uptake of Lipid-Based Emulsions

**Figure 7:**
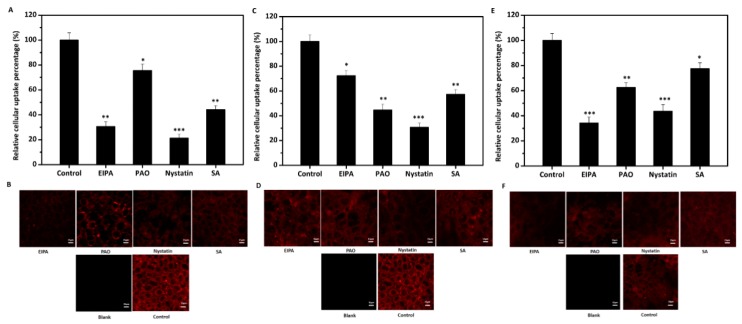
The effects of varying endocytosis inhibitors on the uptake of three lipid-based emulsions (170 nm, 265 nm, and 556 nm, respectively). (**A**,**C**,**E**) Relative uptake of emulsions by cells incubated with DMEM without serum and treated with three specific endocytosis inhibitors and also sodium azide (SA). The control without inhibitors was set as 100%. All data were compared to the control using ANOVA (* *p* < 0.05, ** *p* < 0.01, *** *p* < 0.005). (**B**,**D**,**F**) CLSM of Caco-2 cells grown on glass treated with inhibitors and exposure to three lipid-based emulsions for 4 h. Scale bar indicates 15 μm. The data were expressed as mean ± standard with three independent replicates.

All three various droplet size emulsions (170, 265, and 556 nm) were used for intake mechanism study. Inhibitors of four different endocytosis routes were used to determine mechanism of uptake of lipid-based three different droplet size emulsions. The uptake of the three lipid emulsions was significantly decreased by all four inhibitors (Figure 7). The toxicity of inhibitors at the concentration used on Caco-2 cells were evaluated prior to cellular uptake experiments in this study. Cell viabilities were above 95%, suggesting these inhibitors are nontoxic at the concentration used and the possible inhibition on uptake are not due to cell toxicity.

The endocytosis of droplets is an active transport mechanism and requires energy. In this study, sodium azide [35] decreased emulsions droplets intake by about 50%, 40%, 22%, for 170, 265 and 556 nm emulsions, respectively, indicating that at least some intake is by an endocytosis mechanism (Figure 7). 5-(*N*-Ethyl-*N*-isopropyl) amiloride (EIPA) have been reported to be an effective inhibitor of macropinocytosis [36]. In this study, cellular uptake of all three lipid-based emulsion droplets were decreased approximately 70%, 28%, and 65% for 170, 265 and 556 nm emulsions, respectively with EIPA, indicating macropinocytosis was involved in the internalization and played the most important role for the largest droplet size emulsion (Figure 7). Phenylarsine oxide (PAO) is an inhibitor of clathrin-mediated endocytosis through reacting with vicinal dithiol-containing molecules [37]. The internalization of emulsions was significantly inhibited by about 25%, 55%, and 30% by PAO for 170, 265 and 556 nm emulsions, respectively, suggesting clathrin-mediated endocytosis may also play a vital role in the uptake process. Nystatin is used to inhibit cholesterol-dependent uptake by caveolin- and lipid-raft-mediated endocytosis [38]. The results (Figure 7) clearly showed that Nystatin had highest inhibition effects on both 170 nm and 265 nm emulsions, indicating caveolae/lipid raft-dependent endocytosis may be the most important manner in the internalization of emulsions with relative smaller droplet diameters. Compared to the other two endocytosis routes, clathrin-mediated pathway was relatively less important for 170 and 556 nm emulsions. The results clearly showed that the endocytosis of lipid-based emulsions was size-dependent.

However, previous research of uptake of microwave-produced solid lipid nanoparticles (SLNs) indicated that clathrin-mediated route was the most preferred pathway [29,39]. The difference between our results may be attributed to the differences in surface properties, droplet size, and lipid condition. Our findings illustrate that endocytosis of emulsions is complicated and may be the consequence of the combined action of clathrin, lipid raft/caveolae, and macropinocytosis. Chai and He also demonstrated similar results in the study of the transport mechanisms of SLNs and polymer nanoparticles, respectively [17,37].

### 3.6. Effects of Temperature on the Cellular Uptake of Nanoemulsions

**Figure 8:**
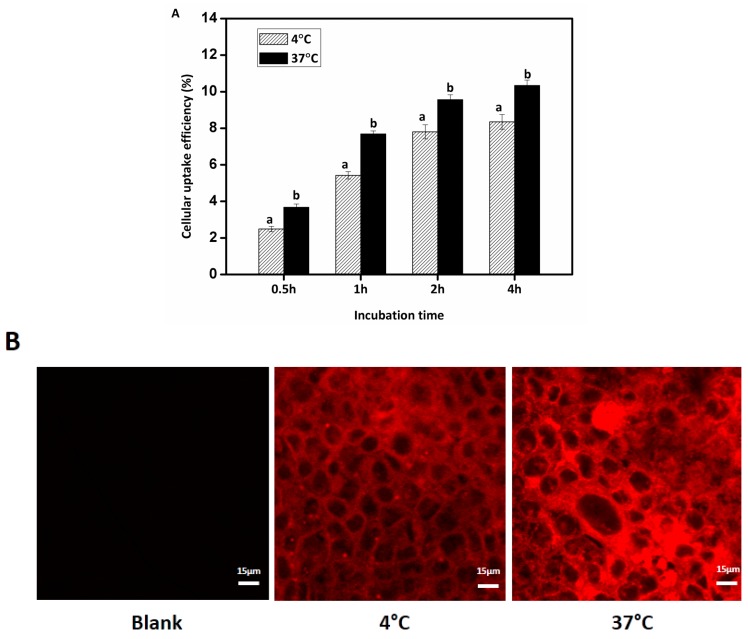
Cellular uptake efficiency (**A**) of lipid-based nanoemulsions at different intervals (0.5, 1, 2, and 4 h) at 4 and 37 °C; CLSM (**B**) of Caco-2 cells after cellular uptake of lipid-based nanoemulsions for 4 h at 4 and 37 °C, respectively. All data were analyzed using ANOVA (Values with different letters at the same time point are significant differences). Scale bar indicates 15 μm. The data were expressed as mean ± standard with three independent replicates.

The smallest diameter emulsion droplets (170 nm) were selected for studies of the effects of temperature on cellular uptake. Nanoemulsion was defined to be a conventional emulsion that contains very small droplets with mean droplet diameter between 20 and 200 nm [40]. Endocytosis is affected by temperature due to effects on membrane fluidity. The cellular uptake of Nile red-labeled nanoemulsions was determined at two different temperatures within 4 h, as depicted in Figure 8. Lipid-based nanoemulsions were obviously internalized into Caco-2 cells at both temperatures as shown by the fluorescence intensity of Caco-2 cells with CLSM. At 37 °C fluorescence intensity was significantly higher than at 4 °C. Quantitative analysis showed that the cellular uptake of nanoemulsions increased gradually with incubation time and the internalization efficiency at 37 °C was greater than at 4 °C. The uptake was 3.7% after 30 min and 9.6% after 2 h incubation at 37 °C. After 4 h incubation, the uptake efficiencies were 10.3% and 8.4% for 37 and 4 °C, respectively (*p* < 0.05). The lower uptake amount at 4 °C was attributed to the low enzyme metabolic activities and poor membrane fluidity. The results showed that the cellular uptake of nanoemulsion is time- and energy-dependent, consistent with the study of Luo et al. who showed that the uptake mechanism of sodium caseinate-encapsulated zein nanoparticles by Caco-2 cell were by an energy-dependent endocytosis [30].

### 3.7. Transport Study

**Figure 9:**
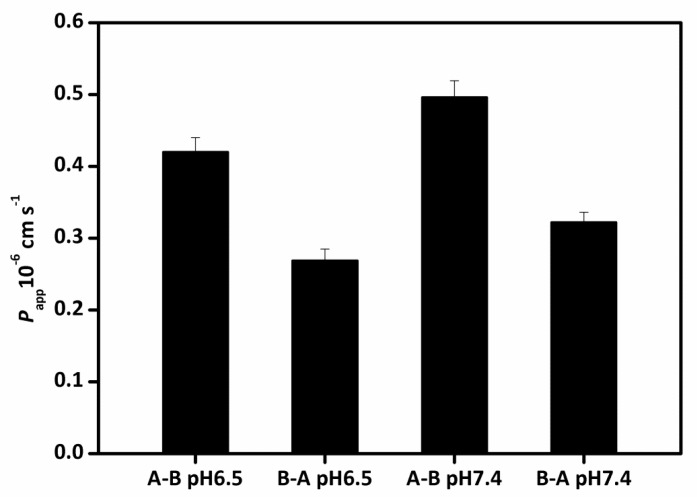
Transport of lipid-based nanoemulsions with Caco-2 cells monolayers Accumulation transport (A) and apparent permeability coefficient (*P*_app_) (B) during 4 h at 37 °C. (Mean droplet size is 170 nm). The data were expressed as mean ± standard with three independent replicates.

The apparent permeation rates (*P*_app_) of lipid-based nanoemulsions were evaluated on both directions (apical (A)-basolateral (B) and B-A) at pH 6.5 and pH 7.4. The use of pH 6.5 donor media was to imitate the pH of the small intestine. For A-B transport, the *P*_app_ values of lipid-based nanoemulsions were 0.50 × 10^−6^ and 0.42 × 10^−6^ cm/s for pH 7.4 and pH 6.5 (Figure 9), respectively, suggesting that the transport of nanoemulsions through the cell is a relatively low rate process. While for B-A transport, the *P*_app_ values were 0.32 × 10^−6^ and 0.27 × 10^−6^ cm/s for pH 7.4 and pH 6.5, respectively. The results showed that the *P*_app_ values at pH 6.5 were lower than that at pH 7.4, consistent with the results in other literature [41]. The efflux ratios *P*_app_ (A-B)/*P*_app_ (B-A) were 1.54 and 1.56, respectively for pH 7.4 and pH 6.5. The (*P*_app_) values for A-B transport were remarkably higher than B-A, indicating the transport of lipid-based nanoemulsions may be an active process and some receptor may be involved in. No appreciably TEER values changes were observed during transport, suggesting cell monolayers kept intact and the structure of tight junctions were not destroyed with nanodroplet transport. The results indicated that nanoemulsions had no cytotoxicity during transport.

## 4. Conclusions

In conclusion, three different droplet size lipid-based emulsions were prepared and the effects of droplet size on cellular toxicity, cellular uptake, uptake mechanism, and transport of emulsions were analyzed. No visible creaming or aggregation were observed for all three emulsions during 30 d storage. The MTT assay of lipid-based emulsions with Caco-2 cells demonstrated that the sodium caseinate emulsions had low cytotoxicity, especially at 2 mg sodium caseinate/mL or less. Positively correlated relationship between smaller mean droplet size and increased cellular uptake was observed. The transport of lipid-based nanoemulsion was an active process. The uptake of the lipid nanoemulsions was by a time- and energy-dependent endocytosis mechanism. The internalization of nanoemulsions was mainly by clathrin- and lipid raft (caveolae)-related pathway as well as macropinocytosis. This study confirmed the consistently reported increased bioavailability of nanoemulsions. The results obtained may provide useful information for the fabrication of nanoemulsions for the efficient delivery of bioactive compounds with low water solubility.

## Figures and Tables

**Table 1:** Inhibitors with different endocytosis functions and the concentrations [17].

Endocytosis Inhibitors	Inhibitor of	Concentrations
EIPA	macropinocytosis pathway	50 μM
PAO	clathrin-mediated endocytosis	10 μM
Nystatin	caveolae/lipid raft-dependent endocytosis	30 μM
Sodium azide	energy-dependent route	1 mg/mL

**Table 2:** Characteristics of three different droplet size lipid-based emulsions (mean ± STD, *n* = 3) ^α^.

Type	Compositions	Initial Emulsion			Emulsion after 30 Days		
		Mean Droplet Size (nm)	PDI	Zeta-Potential	Mean Droplet Size (nm)	PDI	Zeta-Potential
1	10% corn oil, 2% sodium caseinate, 88% distilled water	556.4 ± 3.4a	0.198 ± 0.037	−40.5 ± 1.7a	629.5 ± 2.7a	0.235 ± 0.041	−43.6 ± 1.5a
2	10% corn oil, 2% sodium caseinate, 88% distilled water	265.2 ± 2.5b	0.150 ± 0.025	−41.2 ± 1.4a	283 ± 1.6.5b	0.176 ± 0.017	−41.5 ± 1.2a
3	10% corn oil, 2% sodium caseinate, 88% distilled water	170.3 ± 2.1c	0.113 ± 0.021	−42.3 ± 1.8a	182 ± 2.4c	0.142 ± 0.026	−42.6 ± 1.7a

^α^ Numbers are the mean ± standard deviation of triplicates. All data were analyzed using ANOVA. Values with different letters (a–c) in the same column represent significant difference (*p* < 0.05).

## Abbreviations

SC	sodium caseinate
CLSM	Confocal laser scanning microscopy
BC	beat-carotene
o/w	Oil-in-water
ROS	reactive oxygen species
EIPA	5-(*N*-Ethyl-*N*-isopropyl)amiloride
PAO	phenylarsine oxide
FBS	fetal bovine serum
TER	transepithelial electrical resistance
Dz	mean droplet sizes
PDI	polydispersity index
MTT	3-(4,5-Dimethylthiazol-2-yl)-2,5-diphenyltetrazolium bromide
TEM	Transmission electron microscopy
DMEM	the dulbecco’s modified eagle medium
